# Performance of two rapid antigen tests against SARS-CoV-2 in neighborhoods of socioeconomic vulnerability from a middle-income country

**DOI:** 10.1371/journal.pone.0298579

**Published:** 2024-06-21

**Authors:** Diana Zeballos, Laio Magno, Thais Aranha Rossi, Fabiane Soares, Jony Arrais Pinto Junior, Orlando Ferreira, Carina Carvalho dos Santos, Joice Neves Reis, Thiago S. Torres, Valdilea G. Veloso, Inês Dourado

**Affiliations:** 1 Instituto de Saúde Coletiva, Universidade Federal da Bahia, Salvador, Bahia, Brazil; 2 Departamento de Ciências da Vida, Universidade do Estado da Bahia, Salvador, Bahia, Brazil; 3 Departamento de Estatística, Universidade Federal Fluminense, Niterói, Rio de Janeiro, Brazil; 4 Laboratório de Virologia Molecular, Instituto de Biologia, Universidade Federal do Rio de Janeiro, Rio de Janeiro, Brazil; 5 Departamento de Análises Clínicas e Toxicológicas, Faculdade de Farmácia, Universidade Federal da Bahia, Salvador, Bahia, Brazil; 6 Instituto Nacional de Infectologia Evandro Chagas, Fundação Oswaldo Cruz, Rio de Janeiro, Brazil; Menzies School of Health Research, AUSTRALIA

## Abstract

**Background:**

As new and improved antigen-detecting rapid diagnostic tests for SARS-CoV-2 infection (Ag-RDT) continue to be developed, assessing their diagnostic performance is necessary to increase test options with accurate and rapid diagnostic capacity especially in resource-constrained settings. This study aimed to assess the performance of two Ag-RDTs in a population-based study.

**Methods:**

We conducted a diagnostic accuracy study in neighborhoods with high socioeconomic vulnerability in Salvador-Brazil, including individuals aged ≥12 years old who attended primary health services, between July and December 2022, with COVID-19 symptoms or who had been in contact with a confirmed case. Two Ag-RDTs were compared in parallel using reverse transcription polymerase chain reaction (RT-PCR) as reference standard, the Panbio^TM^ COVID-19 Ag test (Abbott®) and Immuno-Rapid COVID-19 Ag (WAMA Diagnostic®). Sensitivity, specificity, positive (PPV) and negative predictive values (NPV) were calculated.

**Results:**

For the Abbott test the sensitivity was 52.7% (95% CI: 44.3% - 61.0%), specificity 100% (95% CI: 98.7% - 100%), PPV 100% (95% CI: 95.4% - 100%) and NPV 80.4% (95% CI: 75.9% - 84.4%). For the WAMA test, the sensitivity was 53.4% (95% CI: 45.0% - 61.6%), specificity 100% (95% CI: 98.7% - 100%), PPV 100% (95% CI: 95.4% - 100%) and NPV 80.7% (95% CI: 76.2% - 84.6%). Sensitivity for the group with Cycle Threshold (CT) <24 was 82.3% (95%CI: 72.1–90.0, n = 83) for Panbio^TM^ COVID-19 Ag test and 87.3% (95%CI: 77.9–93.8, n = 83) for Immuno-Rapid COVID-19 Ag test.

**Conclusion:**

Sensitivity for both Ag-RDT was lower than reported by manufacturers. In the stratified analysis, sensitivity was higher among those with lower CT values <24. Specificity was high for both rapid antigen tests. Both Ag-RDT showed to be useful for rapid diagnostic of potential cases of COVID-19. Negative results must be assessed carefully according to clinical and epidemiological information.

## Introduction

COVID-19 has become an established and ongoing health issue. In May 2023, after more than three years since the first case of SARS-CoV-2 was identified, the World Health Organization (WHO) declared that COVID-19 is no longer public health emergency of international concern [1]. However, it continues to be a global threat, especially for low- and middle-income countries where the inequities for access to testing, vaccines, and treatments maintain significant burdens and the emergence of new variants also represents a risk for further surges in cases and deaths [2, 3]. Consequently, early diagnoses continue to be fundamental to control the spread of SARS-CoV-2 infection and to prevent new outbreaks. WHO recommendations include mass testing and strategies that expand access to testing for COVID-19 [4].

The identification of viral RNA using real-time reverse transcription polymerase chain reaction (RT-qPCR) was commonly used as a reference standard for diagnosing SARS-CoV-2 infection, especially during the first wave of the pandemic [5]. However, its expanded implementation has been hindered by its cost, the requirement of trained staff, laboratory facilities, and the time it takes to deliver results that delays self-isolation and undermines efforts to contain the disease within communities where early diagnosis to prevent the infection spread is fundamental [6, 7] The early development and implementation of antigen-detecting rapid diagnostic tests for SARS-CoV-2 infection (Ag-RDT) became a fast alternative for diagnosis of this infection, since they are easy to perform, requiring minimal training and yielding results within 15 to 30 minutes [8]. Ag-RDTs have demonstrated their potential to serve as a point-of-care testing option, thus enhancing accessibility and mass testing to improve surveillance, identification of high-risk populations and outbreaks control, particularly within resource-limited settings [9]. The disparity in access to essential tools for mitigating the impact of COVID-19 was evident among low- and middle-income countries. Locally manufactured Ag-RDTs represent a potential advantage, offering an opportunity to enhance accessibility [3].

The analysis of the performance of Ag-RDT for clinical and epidemiological validation was constantly evaluated in different settings since the release of the tests at the first global COVID-19 wave [10], as such studies are essential for adopting these technologies by public health systems. A series of systematic reviews have been conducted to summarize that evidence, observing that sensitivity varied considerably between studies, with consistently high specificities; sensitivity is higher among symptomatic than in asymptomatic individuals with a pooled estimation of 73% vs 54.7% [10]. In Brazil, the accuracy of some of commercialized Ag-RDTs have been evaluated with results of sensitivity lower than reported by its manufacturer varying from 9.8% to 81.1% [11]. As new and improved Ag-RDTs continue to be developed, it is necessary to assess their performance to increase test options with accurate and rapid diagnostic capacity especially in resource-limited settings. In this sense, this study aimed to assess the performance of two Ag-RDTs in a population-based study.

## Methods

### Study design, participants and setting

We conducted a diagnostic accuracy study with data from the “Expansion of testing, quarantine, e-health, and telemonitoring strategies to fight against the COVID-19 pandemic in Brazil (TQT-COVID-19 Study)”, a study conducted in high socioeconomic vulnerability neighborhoods from two Brazilian capital cities. The study protocol has been described elsewhere [12].

For this sub-study, we consecutively enrolled individuals that attended two primary health care services in Salvador between July 11 and December 5, 2022. The participants had to meet the following criteria: age ≥ 12 years, with COVID-19 symptoms (Table 1) or who had been in contact with a confirmed COVID-19 case, regardless of previous vaccination or infection status. Individuals with active nose bleeding, acute facial injuries or trauma were not included in the study.

**Table 1:** COVID-19 symptoms considered in the TQT COVID-19 study. July to December 2022, Salvador-Brazil.

COVID-19 Symptoms	
• Fever	• Shortness of breath
• Dry cough	• Nasal congestion
• Cough with phlegm	• Nausea and vomiting
• Sore throat	• Joint pain
• Coryza	• Muscle pain
• Headache	• Stomachache
• Chills	• Sleep pattern alteration
• Chest pain	• Psychomotor agitation
• Fatigue	• Burning eyes
• Diarrhea	• Lethargy
• Loss of smell	• Skin changes
• Loss of taste	

### Data collection

Two Ag-RDTs approved by the Brazilian Health Regulatory Agency (ANVISA) were compared in parallel: the Panbio^TM^ COVID-19 Ag test (Abbott®) and Immuno-Rapid COVID-19 Ag (WAMA Diagnostic®). Characteristics of the tests provided by the manufacturer are displayed in Table 2. Sample collection was performed prospectively by health professionals following manufacturer instructions. We used RT-qPCR testing as reference standard. Sample collection for both RT-qPCR and Ag-RDTs was carried out on the same day. One nostril was assigned for the Panbio^TM^ COVID-19 Ag test, another nostril for the Immuno-Rapid COVID-19 Ag test, and both nostrils for RT-qPCR, consistently following the same order.

**Table 2:** Characteristics of rapid antigen tests against SARS-CoV-2.

Characteristic	Panbio^TM^ COVID-19 Ag test	Immuno-Rapid COVID-19 Ag
**Manufacturer**	Abbott	WAMA Diagnostic
**Country**	Germany	Brazil
**Antigen detected**	N protein	N protein
**Specimen**	Nasopharyngeal	Nasopharyngeal
**Time to result**	15 minutes	15–20 minutes
**Sensitivity** ^a^	93.3% (95%CI: 83.8–98.2%)	95.8% (95%CI: 91.9–97.8%)
**Specificity** ^a^	99.4% (95%CI: 97.0–100%)	99.6% (95%CI: 97.7–99.95)

CI = Confidence Interval.

^a^As reported by manufacturer.

For RT-qPCR, the swab sample was immersed in an RNA shield medium (Zymo Research) to transport and contain it until extraction. Samples were transported to the reference laboratory on the same day. RNA extraction was performed using the Maxwell® RSC 48 Viral Total Nucleic Acid Purification System (Promega). RT-qPCR reactions were performed using the Allplex SARS-CoV-2 kit (Seegene) according to the manufacturer’s instructions. The RT-qPCR was considered positive when the Cycle threshold (CT) values for E-gene, RdRP-gene, and N-gene were ≤ 40 or the CT values for two genes were under 40. The RT-qPCR was considered negative when the amplification signal was absent. Any other RT-qPCR result was considered inconclusive.

A socio-behavioral questionnaire was applied to individuals who accepted to participate in the study, and collected data on age, gender identity (i.e., cisgender men, cisgender women, transgender men, and transgender women), race (i.e., Black, *Pardo* (mixed race/skin color), Asian, Indigenous, and White), onset of symptoms, COVID-19 vaccination, knowledge, attitudes and practices for COVID-19 prevention.

### Sample size

Using the sample size calculation proposed by Hajian-Tilaki [13] a sample size of 246 individuals would provide 80% of statistical power with a 95% confidence level, considering a sensitivity of 80% reported in a previous systematic review for individuals in the first week of symptoms [10], an expected marginal error of 0.10 and a positive rate of 26% during the TQT study.

### Data analysis

The main characteristics of the population are presented using descriptive statistics. Sensitivity, specificity, positive (PPV) and negative predictive values (NPV) were calculated for each Ag-RDT compared with RT-qPCR with their corresponding 95% confidence interval (95%CI). CT values are inversely proportionate to viral load, with higher CT values indicating lower viral loads, also correlated with the infection’s duration [14, 15]. Complementary analyses were performed comparing sensitivity, specificity, PPV, and NPV according to stratified CT values of the N-gene CT (< 24, 24 ≤ CT ≤ 30, and > 30), as both Ag-RDTs detect the N protein. CT categories were selected based on descriptive statistics of CT values (S1 Table and S1 Fig), and indeterminate CT values for the N gene, were excluded in the stratified analysis. Statistical analysis was conducted using the R project (https://www.r-project.org/)

### Ethical issues

The TQT-COVID-19 study was conducted according to the Brazilian (Resolution CNS no. 466, Brazil, 2012 and Resolution CNS no. 510, Brazil, 2016) and international research ethics guidelines. The study was approved by the Ethics Research Committees of the World Health Organization (Protocol ID: CERC.0128A and CERC.0128B) and for local Brazilian Institutional Review Boards in Salvador (ISC/UFBA: 53844121.4.1001.5030) A written informed consent (WIC) was obtained from participants aged ≥ 18 years. For those < 18 years a written assented consent was fulfilled by them and a WIC from parents or guardians was obtained.

## Results

### Population characteristics

**Fig 1:**
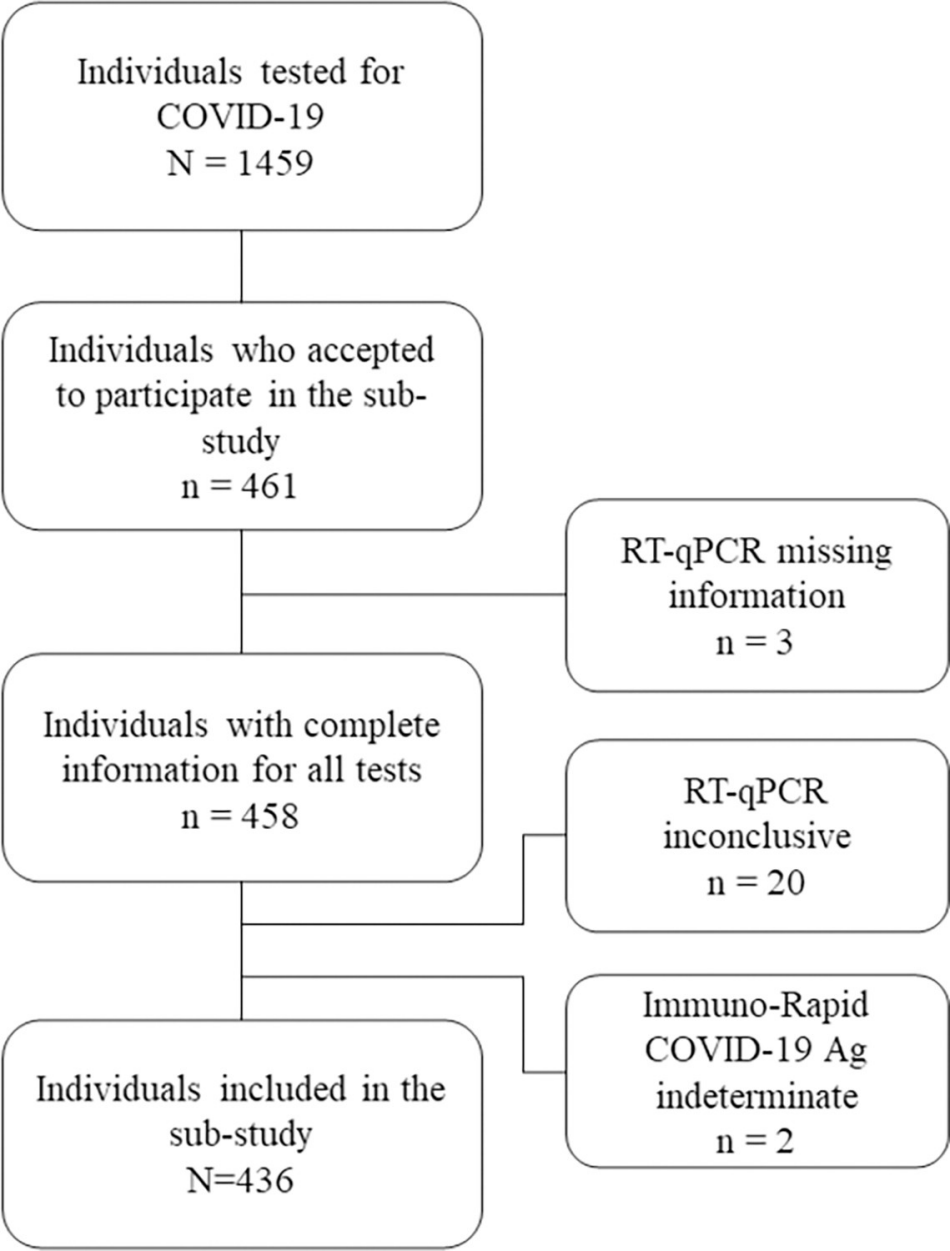
Flow of participants in two sites of the TQT COVID-19 study. July to December 2022, Salvador-Brazil.

A total of 1459 individuals were tested for COVID-19 during the study period with at least one of the study tests. From these, 461 individuals accepted to participate in this sub-study and collected samples. We excluded 24 participants due to: RT-qPCR missing information (n = 3), inconclusive RT-qPCR result (n = 20) and indeterminate result for Immuno-Rapid COVID-19 Ag (n = 2). Thus, we included 436 individuals in this sub-study (Fig 1).

The median age was 41.1 years (range 16 to 91 years), most were cisgender women (68.3%) and *Pardo* (44.0%). More than half of the participants had a contact with a confirmed case of COVID-19 in the previous two weeks (59.1%). The most common symptoms were headache (57.6%), dry cough (44.0%), sore throat (46.1%), fever (29.3%) and nasal congestion (30.9%). Most participants had been boosted for COVID-19 vaccines (77.5%) (Table 3). The prevalence of SARS-CoV-2 infection estimated by RT-qPCR in this sub-study was 33.9% (148/436), and the mean of CT among positives was 25.1 (S1 Table and S1 Fig).

**Table 3:** Characteristics of participants included in the TQT COVID-19 study. July to December 2022, Salvador-Brazil.

Characteristic	n/N	%
**Age**		
10–19 years	14/436	3.2
20–24 years	27/436	6.2
25–59 years	333/436	76.4
>60 years	42/436	9.6
NA	20/436	4.6
**Gender identity**		
Cisgender women	298/436	68.3
Cisgender men	112/436	25.7
Transgender women	2/436	0.5
NA	24/436	5.5
**Race**		
Black	165/436	37.8
*Pardo*	192/436	44.0
White	49/436	11.2
Asian	8/436	1.8
Indigenous	1/436	0.2
NA	21/436	4.8
**Symptoms**		
Headache	232/403	57.6
Coryza	193/403	47.9
Sore throat	182/395	46.1
Dry cough	175/398	44.0
Nasal congestion	121/391	30.9
Fever	115/393	29.3
Muscle pain	93/390	23.8
Fatigue	85/389	21.8
Shortness of breath	69/389	17.7
Chills	58/387	14.9
Cough with phlegm	52/391	13.3
Chest pain	44/386	11.4
Nausea and vomiting	40/385	10.4
Loss of taste	39/382	10.2
Joint pain	39/383	10.2
Burning eyes	39/385	10.1
Diarrhea	30/387	7.7
Loss of smell	28/381	7.3
Sleep pattern alteration	27/385	7.0
Lethargy	22/381	5.8
Skin changes	22/381	5.8
Stomachache	21/382	5.5
Psychomotor agitation	8/383	2.1
None of those	17/381	4.4
**Case contact (two weeks)**		
Yes	247/436	59.1
No	171/436	40.9
NA	18/436	4.1
**Vaccines**		
At least one boosted dose	338/436	77.5
Fully vaccinated	68/436	15.6
Incomplete	6/436	1.4
NA	24/436	5.5

NA, Not Available

### Ag-RDT performance

The Panbio^TM^ COVID-19 Ag test sensitivity was 52.7% (95% CI: 44.3% - 61.0%), specificity 100% (95% CI: 98.7% - 100%), PPV 100% (95% CI: 95.4% - 100%) and NPV 80.4% (95% CI: 75.9% - 84.4%). For the Immuno-Rapid COVID-19 Ag test, the results were sensitivity of 53.4% (95% CI: 45.0% - 61.6%), specificity of 100% (95% CI: 98.7% - 100%), PPV of 100% (95% CI: 95.4% - 100%) and NPV of 80.7% (95% CI: 76.2% - 84.6%). A total of 252 individuals with indeterminate CT results for N-gene were excluded from the stratified analysis. Additionally, 8 participants with missing data for N-gene CT values were excluded from the analysis. In the analysis stratified by CT values, for the group with CT < 24 the sensitivity was 82.3% (95%CI: 72.1–90.0, n = 83) for Panbio^TM^ COVID-19 Ag test and 87.3% (95%CI: 77.9–93.8, n = 83) for Immuno-Rapid COVID-19 Ag test. The sensitivity was lower for the other CT value groups (Table 4). Stratified analysis based on CT values for both the E-gene and RdRp-gene yielded similar results (S2 Table).

**Table 4:** Performance of rapid antigen tests against SARS-CoV-2 overall and stratified by N-gene CT value in TQT COVID-19 study. July to December 2022, Salvador-Brazil.

Tests	n	True positive	False positive	False negative	True negative	Sensitivity	Specificity	PPV	NPV
						% (95%CI)	% (95%CI)	% (95%CI)	% (95%CI)
**Panbio COVID-19 Ag test (Abbott®)**									
**All**	436	78	0	70	288	52.7 (44.3–61.0)	100 (98.7–100)	100 (95.4–100)	80.4 (75.9–84.4)
**RT-qPCR N-Gene CT value** *									
CT <24	83	65	0	14	4	82.3 (72.1–90.0)	100 (88.8–100)	100 (94.5–100)	22.2 (6.4–47.6)
24 ≤ CT ≤ 30	25	9	0	16	y0	36.0 (18.0–57.5)	--	100 (66.4–100)	-- (--)
CT >30	68	3	0	38	27	7.3 (1.5–19.2)	100 (88.8–100)	100 (29.2–100)	41.5 (29.4–54.4)
**Immuno-Rapid COVID-19 Ag (WAMA Diagnostic®)**									
**All**	436	79	0	69	288	53.4 (45.0–61.6)	100 (98.7–100)	100 (95.4–100)	80.7 (76.2–84.6)
**RT-qPCR CT value Gen N** *									
CT <24	83	69	0	10	4	87.3 (77.9–93.8)	100 (88.8–100)	100 (94.8–100)	28.6 (8.4–58.1)
24 ≤ CT ≤ 30	25	8	0	17	0	32.0 (14.9–53.5)	--	100 (63.6–100)	--
CT >30	68	1	0	40	27	2.4 (0.1–12.9)	100 (88.8–100)	100 (2.5–100)	40.3 (28.5–53.0)

RT-qPCR = reverse transcription polymerase chain reaction; CT = Cycle Threshold; PPV = Positive Predictive Value; NPV = Negative Predictive Value.

* Indeterminate CT values for the N gene were excluded in the stratified analysis

## Discussion

We assessed the performance of two Ag-RDTs among individuals with access to two primary healthcare centers located in highly vulnerable neighborhoods in Salvador. We observed that the overall sensitivity was low for both rapid antigen tests, and lower than reported by manufacturers. However, in the stratified analysis, sensitivity was higher among those with CT values <24 and lower among those with CT values above or equal to 24. Specificity was high for both rapid antigen tests. It is worth noting that CT value differences and sensitivity variations are associated with viral load, which tends to be high during the first week of infection. Besides, both Ag-RDTs utilize the virus’s nucleocapsid protein (N) as the target. This protein is commonly chosen due to its minimal variation, which could otherwise decrease sensitivity in the presence of mutations [7].

For Panbio^TM^ COVID-19 Ag test our findings were similar to those observed in previous studies. A study conducted in Brazil included individuals with moderate or mild COVID-19 symptoms who were admitted in tertiary hospitals found a sensitivity of 60.0% (95%CI: 45.9–73.0%), with an increase in sensitivity among patients within seven days since the onset of symptoms [11]. A study conducted in Kenya found a sensitivity of 46.6% (95%CI: 42.4–50.9%), with higher sensitivity among symptomatic individuals (60.6%), among who’s onset of symptoms was < 5 days (67.8%) and among symptomatic individuals with CT values equal or lower than 30 (87.0%) [16]. Finally, a systematic review found 24 studies assessing the mentioned test with a pooled sensitivity of 74.8% (95%CI 67.6–80.8) among symptomatic and 14 studies reporting sensitivities among asymptomatic with a pooled estimate of 56.9% (95% CI: 42.8–69.9) [10].

Regarding Immuno-Rapid COVID-19 Ag test, to the best of our knowledge, this is the first study assessing its performance in a point of care testing at community-based health services. The performance was similar to Panbio^TM^ COVID-19 Ag test in comparison with RT-PCR. Overall Immuno-Rapid COVID-19 Ag test detected one case more than Panbio^TM^ COVID-19 Ag test. While overall trends in sensitivity were similar for both rapid tests when stratified by CT category, the Immuno-Rapid COVID-19 Ag test displayed slightly higher sensitivity in the CT<24 category compared with PanbioTM COVID-19 Ag test, and the inverse was true for CT categories >24. Although both tests exhibited performance levels lower than those recommended by WHO, they still fell within the average range of available tests [17].

This study has some potential limitations. First, we were unable to conduct an analysis stratified by presence of symptoms or by the number of days of symptoms due to the high number of missing data in both variables. However, we were able to conduct the analysis using CT values. Since our study was conducted in 2022, a period in which the Omicron variant and its sub-variants were circulating in Salvador, our results must be interpreted with caution, as there is still limited knowledge about changes in the sensitivity of tests with Omicron versus other variants. Nonetheless, a study carried out in the UK found that the performance of rapid antigen test devices was similar in different scenarios of vaccination and regarding the viral variants [18].

In conclusion, we demonstrated that a locally manufactured test with lower costs exhibited comparable performance to a test that has been in use since the initial year of the COVID-19 pandemic and against RT-qPCR. This test offers a viable alternative in resource-limited settings and holds significance in environments lacking proper laboratory facilities for COVID-19 diagnosis. Given the persistent public health concern posed by COVID-19 and the role of testing in identifying outbreaks, the adoption of accessible and effective tests should be considered while acknowledging their limitations.

## Supporting information

S1 Fig Boxplot of cycle threshold values for the three genes, by RT- qPCR results. (DOCX)

S1 Table Description of cycle threshold values for the three genes evaluated for RT-qPCR. (DOCX)

S2 Table Performance of rapid antigen tests against SARS-CoV-2 overall and by E-gene and RdPd-gene CT values in TQT COVID-19 study. July to December 2022, Salvador-Brazil.
(DOCX)
