# Supplementary figures and images for: Performance of two rapid antigen tests against SARS-CoV-2 in neighborhoods of socioeconomic vulnerability from a middle-income country
Source: PLoS One. 2024 Jun 21;19(6):e0298579. doi: 10.1371/journal.pone.0298579 (PMC11192360; doi:10.1371/journal.pone.0298579)

**
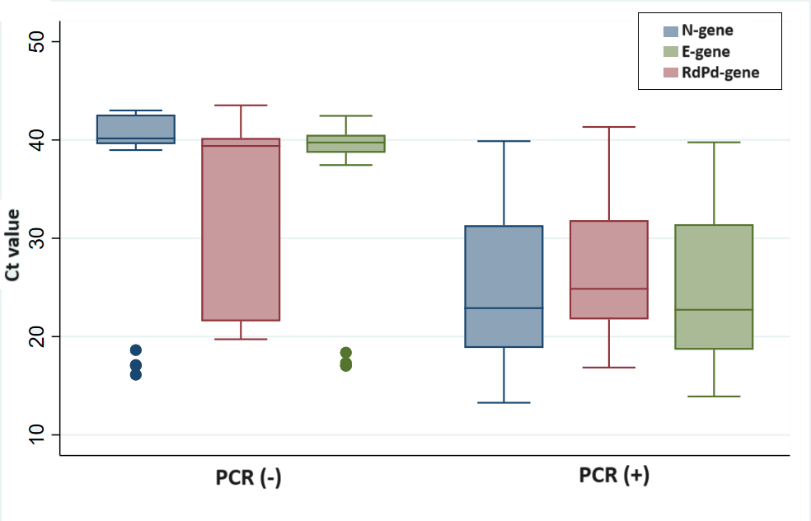
**

**S1 Fig. Boxplot of cycle threshold values for the three genes, by RT- qPCR results.**

Supplement: S1 Fig — (DOCX) [file pone.0298579.s001.docx]
