# Supplementary material for: Performance of two rapid antigen tests against SARS-CoV-2 in neighborhoods of socioeconomic vulnerability from a middle-income country
Source: PLoS One. 2024 Jun 21;19(6):e0298579. doi: 10.1371/journal.pone.0298579 (PMC11192360; doi:10.1371/journal.pone.0298579)
Supplement: S1 Table — (DOCX) [file pone.0298579.s002.docx]

**S1 Table. Description of cycle threshold values for the three genes evaluated for RT-qPCR.**

| **CT values** | **Mean** | **Median** | **Percentile 25** | **Percentile 75** |
| --- | --- | --- | --- | --- |
| **Overall** |  |  |  |  |
| N-gene | 27.4 | 25.5 | 19.2 | 36.4 |
| E-gene | 26.9 | 24.8 | 18.8 | 36.0 |
| RdPd-gene | 27.7 | 25.0 | 21.7 | 33.6 |
|  |  |  |  |  |
| **Positive cases** | |  |  |  |
| N-gene | 25.1 | 22.9 | 18.4 | 31.3 |
| E-gene | 25.0 | 22.7 | 18.7 | 31.4 |
| RdPd-gene | 27.0 | 24.8 | 21.7 | 31.8 |
|  |  |  |  |  |
| **Negative cases** | |  |  |  |
| N-gene | 37.9 | 40.2 | 39.6 | 42.6 |
| E-gene | 36.7 | 39.7 | 38.7 | 40.5 |
| RdPd-gene | 34.5 | 39.4 | 21.5 | 40.2 |
