# Supplementary material for: Performance of two rapid antigen tests against SARS-CoV-2 in neighborhoods of socioeconomic vulnerability from a middle-income country
Source: PLoS One. 2024 Jun 21;19(6):e0298579. doi: 10.1371/journal.pone.0298579 (PMC11192360; doi:10.1371/journal.pone.0298579)
Supplement: S2 Table — July to December 2022, Salvador-Brazil. (DOCX) [file pone.0298579.s003.docx]

**S2 Table.** **Performance of rapid antigen tests against SARS-CoV-2 overall and by E-gene and RdPd-gene CT values in TQT COVID-19 Study. July to December 2022, Salvador-Brazil**

| **Testes** | **N** | **True positive** | **False positive** | **False negative** | **True negative** | **Sensitivity % (95%CI)** | **Specificity % (95%CI)** | **PPV % (95%CI)** | **NPV % (95%CI)** |
| --- | --- | --- | --- | --- | --- | --- | --- | --- | --- |
|  |  |  |  |  |  |  |  |  |  |
| **Panbio COVID-19 Ag test (Abbott®)** | | | | | | | | | |
| **E-gene** |  |  |  |  |  |  |  |  |  |
| CT <24 | 82 | 65 | 0 | 13 | 4 | 83.3 (73.2 - 90.8) | 100 (39.8 - 100) | 100 (94.5 - 100) | 23.5 (6.8 - 49.9) |
| 24 ≤ CT ≤ 30 | 29 | 10 | 0 | 19 | 0 | 34.5 (17.9 - 54.3) | -- | 100 (69.1 - 100) | -- |
| CT >30 | 62 | 2 | 0 | 36 | 24 | 5.3 (0.6 - 17.7) | 100 (85.7 - 100) | 100 (15.8 - 100) | 40.0 (27.6 - 53.5) |
| **RdPd-gene** |  |  |  |  |  |  |  |  |  |
| CT <24 | 63 | 51 | 0 | 8 | 4 | 86.4 (75.0 - 94.0) | 100 (39.8 - 100) | 100 (93.0 - 100) | 33.3 (9.9 - 65.1) |
| 24 ≤ CT ≤ 30 | 37 | 22 | 0 | 15 | 0 | 59.5 (42.1 - 75.2) | -- | 100 (84.6 - 100) | -- |
| CT >30 | 48 | 3 | 0 | 36 | 9 | 7.7 (1.6 - 20.9) | 100 (66.4 - 100) | 100 (29.2 - 100) | 20.0 (9.6 - 34.6) |
|  |  |  |  |  |  |  |  |  |  |
| **Immuno-Rapid COVID-19 Ag (WAMA Diagnostic®)** | | | | | | | | | |
| **E-gene** |  |  |  |  |  |  |  |  |  |
| <24 | 82 | 63 | 0 | 12 | 4 | 84.0 (73.7 - 91.4) | 100 (39.8 - 100) | 100 (94.3 - 100) | 25.0 (7.3 - 52.4) |
| 24 ≤ CT ≤ 30 | 29 | 12 | 0 | 17 | 0 | 41.4 (23.5 - 61.1) | -- | 100 (73.5 - 100) | -- |
| >30 | 62 | 0 | 0 | 38 | 24 | 0 (0.0 - 9.2) | 100 (85.7 - 100) | -- | 38.7 (26.6 - 51.9) |
| **RdPd-gene** |  |  |  |  |  |  |  |  |  |
| <24 | 63 | 53 | 0 | 6 | 4 | 89.8 (79.2 - 96.2) | 100 (38.8 - 100) | 100 (93.3 - 100) | 40.0 (12.2 - 73.8) |
| 24 ≤ CT ≤ 30 | 37 | 22 | 0 | 15 | 0 | 59.5 (42.1 - 75.2) | -- | 100 (84.6 - 100) | -- |
| >30 | 48 | 3 | 0 | 36 | 9 | 7.7 (1.6 - 20.9) | 100 (66.4 - 100) | 100 (29.2 - 100) | 20.0 (9.6 - 34.6) |
